# A simple and inexpensive haemozoin-based colorimetric method to evaluate anti-malarial drug activity

**DOI:** 10.1186/1475-2875-11-272

**Published:** 2012-08-09

**Authors:** Tran Thanh Men, Nguyen Tien Huy, Dai Thi Xuan Trang, Mohammed Nasir Shuaibu, Kenji Hirayama, Kaeko Kamei

**Affiliations:** 1 Department of Biology, College of Natural Science, Cantho University, Cantho City, Vietnam; 2 Department of Biomolecular Engineering, Kyoto Institute of Technology, Matsugasaki, Sakyo-ku, Kyoto, 606-8585, Japan; 3 Department of Immunogenetics, Institute of Tropical Medicine (NEKKEN), Nagasaki University, Nagasaki, Japan; 4 Global COE program, Nagasaki University, Nagasaki, Japan

**Keywords:** Anti-malarial, Assay, Haemozoin, Malaria

## Abstract

**Background:**

The spread of drug resistance in malaria parasites and the limited number of effective drugs for treatment indicates the need for new anti-malarial compounds. Current assays evaluating drugs against *Plasmodium falciparum* require expensive materials and equipment, thus limiting the search for new drugs, particularly in developing countries. This study describes an inexpensive procedure that is based on the advantage of a positive correlation between the haemozoin level of infected erythrocytes and parasite load.

**Methods:**

The relationship between parasitaemia and the haemozoin level of infected erythrocytes was investigated after converting haemozoin into monomeric haem. The 50% inhibitory concentration (IC_50_) values of chloroquine, quinine, artemisinin, quinidine and clotrimazole against *P. falciparum* K1 and 9A strains were determined using the novel assay method.

**Results:**

The haemozoin of parasites was extracted and converted into monomeric haem, allowing the use of a colorimeter to efficiently and rapidly measure the growth of the parasites. There was a strong and direct linear relationship between the absorbance of haem converted from haemozoin and the percentage of the parasite (R^2^ = 0.9929). Furthermore, the IC_50_ values of drugs were within the range of the values previously reported.

**Conclusion:**

The haemozoin-based colorimetric assay can be considered as an alternative, simple, robust, inexpensive and convenient method, making it applicable in developing countries.

## Background

Malaria is more than just a problem for tropical countries, it also is a major global public health concern. Annually, there are approximately 300 million new malaria infections and millions of deaths worldwide due to malaria [1,2]. Because a vaccine for malaria is not available, chemotherapy is the main treatment. However, the rapid spread of resistance to current quinoline anti-malarials has made malaria a major global and important problem. In addition, artemisinin, from a Chinese herb (*Qinghaosu*) that has been used in the treatment of fevers for more than a thousand years, is now considered an essential component of artemisinin-based combination therapy against drug-resistant malaria [3,4]. However, the malaria parasites have recently been found to be resistant to artemisinin [5-7]. The alarming spread of drug resistance and the limited number of effective drugs for treatment indicates how important it is to find new anti-malarial compounds.

For decades, the anti-malarial activity of a drug has been measured *in vitro* by quantifying the uptake of radioactive substrates by a parasite as a measure of growth and viability in the presence of the test drug [8,9]. Although several *in vitro* methods exist, the ^3^H-hypoxanthine method [8] is a popular test for novel anti-malarial drugs, but it is labelled with radiation that presents a potential risk to safety, and it relies on relatively expensive radio-isotopes and includes multistep procedures that become increasingly problematic and impractical when the incidence of testing is increased. The other methods, including the PicoGreen [10,11] and the SYBR Green I [12,13] methods, are also considered to be expensive approaches regarding equipment and chemicals. In addition, there are other methods that are based on enzymatic reaction and/or antibodies that specifically detect the presence of histidine-rich protein II or parasite lactate dehydrogenase [14-16]. However, these methods involve multiple complex steps that are too expensive for developing countries, which makes them ill suited for screening potential anti-plasmodial drugs.

During the development and proliferation stage in host erythrocytes, the malaria parasites degrade haemoglobin for use as a major source of amino acids. This is accompanied by the release of free haem. With haem as a prosthetic group of haemoglobin, the iron is in the ferrous state, but free haem loses one electron and assumes the ferric state. This ferric haem could be oxidatively active and toxic to both the host cells and malaria parasites, and can even cause parasite death. Moreover, due to the absence of haem oxygenase, the parasite is unable to cleave haem into an open-chain free haem, which is necessary for cellular excretion [17]. To protect itself, it is necessary for the parasite to convert haem to non-toxic metabolites. Principally, the parasite detoxifies free haem via neutralization with histidine-rich protein 2 [18,19] and degradation with reduced glutathione [20,21], but mostly with crystallization into haemozoin, which is a water-insoluble malarial pigment produced in the food vacuole [19,22]. Therefore, a simple and inexpensive *in vitro* assay was developed based on the colorimetric quantification of haemozoin in infected red blood cells to evaluate the anti-malarial activity of drugs.

## Methods

### Materials

Chloroquine diphosphate, quinine sulphate, primaquine, clotrimazole, haemin chloride (haem), RPMI 1640 medium, hypoxanthine, and gentamycin were purchased from Sigma Aldrich Chemical Company (Tokyo, Japan). Albumax II (Gibco), and the other chemicals used in the present study were of a high grade. *Plasmodium falciparum* K1 (chloroquine resistant) and 3D7-9A (chloroquine susceptible) strains [23] were provided from Dr Osamu Kaneko and Dr Shusuke Nakazawa, respectively, from the Institute of Tropical Medicine, Nagasaki University, Japan.

### *Plasmodium* cultivation

*Plasmodium falciparum* K1 and 9A strains were maintained *in vitro* with continuous culture according to a previously described method with a slight modification [24]. The culture medium consisted of RPMI 1640 supplemented with 0.025 mg/ml gentamicin, 0.01 mM hypoxanthine, 23.8 mM NaHCO_3_, 11 mM glucose and 0.5% albumax II, and adjusted to a pH of 7.3 to 7.4. The parasite was cultured and maintained in a tissue culture flask with complete culture medium containing 5% human erythrocytes. The parasite density was maintained at about 1.5% parasitaemia under an atmosphere of an AnaeroPack sachet (Mitsubishi Gas Chemical Company Inc, Tokyo, Japan) to create 20% CO_2_ and remove O_2_ (<0.1%) 37°C [25]. Every two days, infected erythrocytes were transferred into fresh medium containing 5% human erythrocyte. The level of parasitaemia was determined by light microscopy on a Giemsa-stained thin blood smear, and parasitized erythrocytes were diluted when parasitaemia was higher than 5% in erythrocytes contained at 5% in culture medium, in order to lower parasitaemia and allow continuous growth. Parasite culture was diluted with fresh uninfected erythrocytes and culture medium to achieve a starting parasitaemia of 2% and a haematocrit of 5%. This final parasite culture was immediately used for anti-malarial assay.

### The relationship between parasitaemia and haemozoin level

A culture of the *P. falciparum* K1 strain was serially diluted with uninfected erythrocytes in complete medium to yield a haematocrit of 5% and parasitaemia ranging from 0 to 10%. The serial culture containing 200 μl was prepared independently in triplicate in microtubes, followed by the addition of 800 μl of 2.5% sodium dodecyl sulphate in 0.1 M sodium bicarbonate pH 8.8, then the samples were mixed at room temperature for 15 min. After centrifugation at 13,000 rpm for 10 min, the supernatant was removed. The pellet was washed twice with 800 μl of 2.5% sodium dodecyl sulphate in 0.1 M sodium bicarbonate _(_pH 8.8), then 200 μl of 5% sodium dodecyl sulphate was added to 50 mM NaOH to convert the haemozoin into haem. After incubation at room temperature for 30 min, the sample (200 μl) was transferred to a 96-well microplate and scanned at 405/750 nm (A_405 nm_ minus A_750 nm_) using an IMark microplate reader (Bio-Rad). After the background absorbance of haemozoin was purified of uninfected erythrocytes (5% haematocrit) then subtracted, the amount of haemozoin in the infected erythrocytes was presented as the absorbance at 405/750 nm and then plotted against parasitaemia.

### Evaluating the anti-malarial activity of drugs using the haemozoin-based spectrophotometric method

The *P. falciparum* K1 and 9A strains were used to evaluate the anti-malarial activity of quinine, chloroquine, pyrimethamine, artemisinin and clotrimazole by using the haemozoin-based spectrophotometric method. Stocks of drugs were prepared in dimethyl sulphoxide or phosphate buffer saline (for chloroquine) and were then serially diluted with complete culture medium. To each well of a microplate, 10 μl of serially diluted drug solution was added into 200 μl of final asynchronous parasite culture. Dimethyl sulphoxide or phosphate buffer saline were also tested by adding a similar amount to control wells. The microplates were cultured 72 hr under the conditions described above. The haemozoin of infected erythrocytes was extracted, purified, and quantified, as described above. The 50% inhibitory concentration (IC_50_) value was calculated by non-linear fitting of the absorbance at 405/750 nm against the logarithm of the drug concentration using the GraphPad Prism 5 (GraphPad Software, Inc., San Diego, CA, USA). Sigmoidal doses with variable slope models were used with the following equation:

(1) $$y=\min+\frac{\max-\min}{1+10^{\left(\left\{\log IC_{50}-x\right\}\times Hill\;slope\right)}}$$

where y is the absorbance at 405/750 nm; x is the logarithm of the drug concentration, min is the absorbance at 405/750 nm measured at time zero (starting point of assay), and max is the maximal absorbance of a particular drug. The Hill slope is the steepness of the curve. The logarithm of the concentration at zero was defined at 2 log lower than the lowest concentration of a particular drug.

## Results and discussion

### Relationship between the absorbance of the haem content of haemozoin and parasitaemia

**Figure 1:**
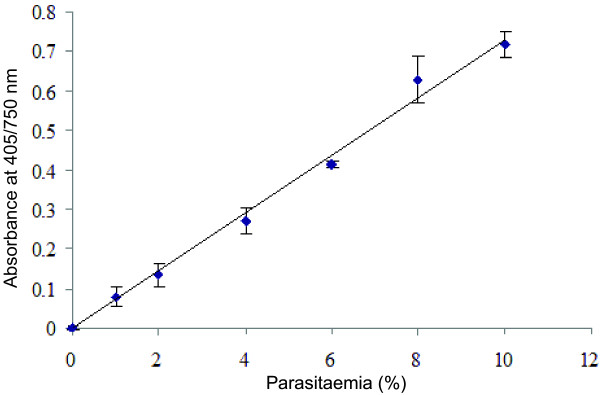
**The linear relationship between the haemozoin level of a parasite and parasitaemia.** Haemozoin concentration of infected erythrocytes is presented by the absorbance at 405/750 nm of monomeric haem after conversion from haemozoin using an NaOH solution. Absorption values (means ± standard errors of triplicate wells) are plotted against parasitaemia. A well correlated, linear relationship (R^2^ = 0.9929) is strong evidence of the sensitivity of the method.

The relationship between the haemozoin amount in a parasitized erythrocyte and parasitaemia was revealed by measuring the absorbance of the haem content of the haemozoin obtained from the parasitized erythrocytes after degradation to monomer haem. As shown in Figure 1, the absorption of converted haem showed a direct and linear correlation with the level of parasitaemia. Low and unsynchronized parasitaemia (about 1%) could be detected using this haemozoin-based colorimetric method. The results indicate that this novel assay of parasites is applicable for monitoring parasite growth and for screening new anti-malarial compounds.

### Haemozoin-based colorimetric assay to determine the IC_50_ values

**Figure 2:**
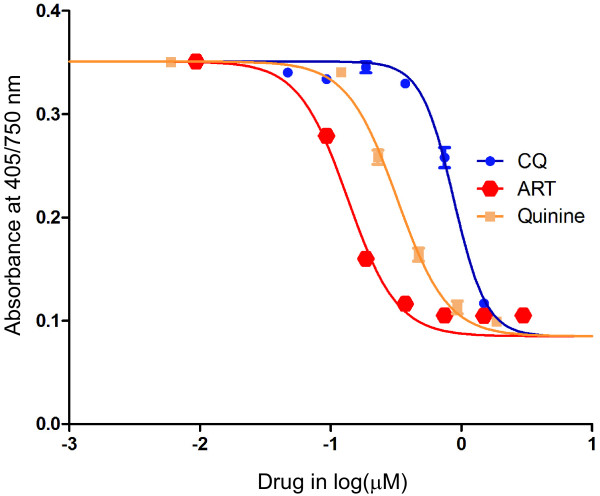
**Representative dose responses for chloroquine (CQ), artemisinin (ART) and quinine against the *Plasmodium falciparum* K1 strain.** Parasite growth after incubation of parasitized erythrocytes for 72 hr with a drug was measured using a haemozoin-based colorimetric method. The symbols and error bars are the average absorption values at 405/750 nm and standard deviations in triplicate, respectively. The sigmoidal dose response model with a variable slope is the best fit to the data.

The IC_50_ values of some anti-malarial drugs were determined with a dose-response experiment using a haemozoin-based colorimetric assay. The result showed that increasing the concentration of anti-malarial drugs resulted in a decreased absorbance at 405/750 nm (Figure 2). The data was best fitted by a typical sigmoidal dose-response model with a variable slope (four parameters) that agreed well with previous reports [26,27].

Table 1 summarizes the results of the assay to determine IC_50_ using the haemozoin-based colorimetric method. The *P. falciparum* K1 strain was primarily observed for quinine, chloroquine, clotrimazole, pyrimethamine and artemisinin, with IC_50_ values of 0.258, 0.873, 0.805, 23.03, and 0.139 μM, respectively. For the *P. falciparum* 9A strain, pyrimethamine was not evaluated, and the IC_50_ values were 0.398, 0.132, 1.67 and 0.483 μM in succession for quinine, chloroquine, clotrimazole and artemisinin, respectively. The IC_50_ values for quinine, chloroquine, clotrimazole, and pyrimethamine were in a range that was similar to those observed in previous reports [28,29]. On the other hand, the IC_50_ values for artemisinine were higher than previous reports, probably due to asynchronous cultures, ring stage-specific target of artemisinine, accumulation of released hemozoin in the continuous cultures, or several rounds of continuous cultures and cloning of parasite strains in our laboratories. Therefore, further studies are required to compare the novel assay with recent developed methods to validate the accuracy in the screening new antimalarial compounds [30]. Another limitation of the novel method is that it is not easily adaptable for a high throughput screening of anti-malarial drug candidates, which is under-developed using 96-well filter plates [31].

**Table 1:** ** *In vitro* anti-malarial activities of drugs against chloroquine-susceptible (9A) and -resistant (K1) strains of *Plasmodium falciparum* **

**Drugs**	**Mean IC**_**50**_**and 95% CI (μM)**	
	** *P. falciparum* K1 strain**	** *P. falciparum* 9A strain**
Quinine	0.258 (0.242 - 0.275)	0.398 (0.307 - 0.516)
Chloroquine	0.873 (0.824 - 0.926)	0.132 (0.082 - 0.211)
Clotrimazole	0.805 (0.689 - 0.939)	1.67 (1.18 – 2.37)
Pyrimethamine	23.03 (18.36 - 28.90)	Not done
Artemisinin	0.139 (0.124 - 0.155)	0.483 (0.376 - 0.622)

Parasitized red blood cells were incubated with different concentrations of drugs for 72 hr and parasite growth was evaluated using the haemozoin-based colorimetric method. The IC_50_ and its 95% confidence interval (95% CI) were calculated from the concentration-response curve of the haemozoin level *vs* the log concentration of a drug

In recent years, the number of laboratories, diagnosis centres and research institutes has risen in developing countries. However, most of them lack the modern equipment and expensive chemicals to apply new methods for screening anti-malarial candidates. In addition, some methods have potential risks of toxicity, so it is prudent to wear disposable gloves at all times when proceeding. Another obstacle is that many laboratories lack the facilities to treat toxic contamination before the toxin is discarded in the environment. The novel anti-malarial assay is safe, non-expensive and easy to apply in laboratories.

## Conclusions

The standard curve obtained in this study was strongly linear between the absorbance of monomeric haem converted from haemozoin and the percentage of parasitaemia. The IC_50_ values of chloroquine and quinine obtained from the haemozoin-based colorimetric method are similar to other methods. Even though this report describes specific conditions, the current experiment has introduced an assay that is adaptable to a wide range of conditions. The results also show that using this method has several advantages over using current methods. First, the method is fast and is based on a simple technique that uses a microplate reader, which is available in most laboratories. Second, the assay is based on inexpensive chemicals with no requirement of cold storage. Last but not least, the assay of the inhibition of *P. falciparum* growth using a haemozoin-based colorimetric method is feasible, reproducible, non-toxic, and more convenient than other assays, which makes it particularly useful for developing countries in the screening of novel anti-plasmodials, as a useful high throughput screening method for anti-malarial drug candidates.

## Competing interests

The authors declare that they have no competing interests.

## Authors’ contributions

NTH and KK developed the idea for the project. TTM and NTH conceived and designed the experiments. TTM, NTH and MNS carried out the laboratory work. TTM, NTH, DTXT, KH, and KK analysed and interpreted the data. TTM, NTH, MNS, KH and KK contributed reagents/materials/analysis tools. . TTM, NTH, DTXT and KK wrote the paper. All authors had full access to all data in the study, read and approved the manuscript.
